# Bronchial asthma: what is different in patients with and without chronic cough?

**DOI:** 10.36416/1806-3756/e20250242

**Published:** 2026-03-05

**Authors:** Dina Visca, Francesco Ardesi, Denise Rossato Silva, Patrizia Pignatti, Marco Vanetti, Martina Zappa, Rosella Centis, Giovanni Battista Migliori, Antonio Spanevello

**Affiliations:** 1 Dipartimento di Medicina e Chirurgia, Università dell’Insubria, Varese, Italia.; 2 Dipartimento di Medicina e Riabilitazione Cardiorespiratoria, Istituti Clinici Scientifici Maugeri IRCCS, Tradate, Italia.; 3 Faculdade de Medicina, Universidade Federal do Rio Grande do Sul - UFRGS - Porto Alegre (RS) Brasil.; 4 Servizio di Allergia e Immunologia, Istituti Clinici Scientifici Maugeri IRCCS, Pavia, Italia.; 5 Servizio di Epidemiologia Clinica delle Malattie Respiratorie, Istituti Clinici Scientifici Maugeri IRCCS, Tradate, Italia.

**Keywords:** Asthma, Chronic cough, Inflammation, Delivery of health care

## Abstract

**Objective:**

Chronic cough (CC)-i.e., cough lasting for ≥ 8 weeks- is a common and burdensome symptom in asthma patients; however, its pathophysiological mechanisms and clinical relevance remain unclear. We sought to investigate clinical, functional, and inflammatory differences between asthma patients with chronic cough (ACC) and those without. (ANCC)

**Methods:**

We included 344 patients with moderate-to-severe asthma evaluated at a referral center from 2017 to 2024. Clinical data, lung function, inflammatory biomarkers, and health resource utilization were compared between ACC and ANCC. Further analyses stratifying patients by sputum eosinophil percentage (≥ 3% vs. < 3%) and CC were also performed. Multivariate logistic regression was used to identify factors independently associated with chronic cough.

**Results:**

ACC (n = 124; 36%) had significantly worse asthma control (ACQ: p = 0.001), as well as higher number of exacerbations (p = 0.011), health care utilization, (p < 0.0001) and treatment dose of inhaled corticosteroids, (p = 0.004) when compared with ANCC. Despite similar lung function and inflammatory biomarkers, dyspnea, nocturnal symptoms, Cumulative Illness Rating Scale scores, and health care use were independently associated with CC. Further stratifications by sputum eosinophil percentage and CC revealed that ACC experienced a greater disease burden, regardless of whether sputum eosinophil percentage was ≥ 3% or < 3%.

**Conclusions:**

CC identifies a subgroup of asthma patients with poorer disease control, increased health care needs, and distinct clinical profiles, independently of airway inflammation. Routine recognition of cough as a symptom in asthma assessment may guide referral and personalized management.

## INTRODUCTION

Asthma is a chronic respiratory disease that is usually characterized by variable respiratory symptoms and airway inflammation.^1^ It is considered a global health problem, affecting approximately 300 million people worldwide.^2^ A significant number of asthma patients continue to experience uncontrolled symptoms and exacerbations, despite improvements in disease management, causing higher health care utilization and socioeconomic burden^3^ and also interfering with the daily life of patients, including work, education, and family.^1^ Symptoms are closely related to medical resource utilization, including unscheduled visits to physicians, emergency room visits, and hospitalizations.^4^ From the perspective of patients, cough appears to be one of the most troublesome symptoms.^5^

Cough is a forced expulsive mechanism that temporarily alters the physiological respiratory pattern.^6^ However, when pathologically severe and persistent, cough is a frequent and debilitating complaint,^7^ ^,^ ^8^ affecting approximately 10% of the adult population worldwide.^9^ Cough is defined as chronic when lasting for ≥ 8 weeks.^6^ Because of its importance in terms of symptom burden and because cough is one of the most common reasons for seeking medical attention,^10^ there has been an increasing focus on cough in asthma. 

Asthma appears to be one of the most common causes of chronic cough.^11^ Different categories of asthma have been distinguished based on the presence of cough: classic asthma; cough-variant asthma; and cough-predominant asthma. Classic asthma is characterized by variable episodes of shortness of breath, chest tightness and wheezing, with mild cough or no cough, whereas cough- variant asthma presents cough as the only symptom of asthma without airflow limitation^12^ and cough-predominant asthma is characterized by persistent cough as the predominant symptom associated with mild wheezing and/or dyspnea.^13^

The role of chronic cough as a symptom in asthma patients has yet to be fully investigated, and agreement on association between airway inflammation and chronic cough in asthma patients is lacking.^14^ ^,^ ^15^ The aim of our study was to compare clinical, functional, and inflammatory characteristics between moderate-to-severe asthma patients with cough for ≥ 8 weeks (ACC) and those without (ANCC). Our hypothesis was that chronic cough in patients with moderate-to-severe asthma is associated with worse disease control, worse lung function, and different airway and/or systemic inflammation. 

## METHODS

### Patients and study design

This was a retrospective observational cross-sectional study conducted at the *Istituti Clinici Scientifici Maugeri*, a referral clinic located in the city of Tradate, Italy). The clinic is specialized in diagnosis and management of asthma patients. All asthma outpatients undergo clinical, physiological, biological, and serological evaluation in order to phenotype asthma for clinical management. Of the patients evaluated in the 2017-2024 period, those who were diagnosed with moderate-to-severe asthma in accordance with the 2024 GINA criteria^1^ were consecutively included in the present study. The study was conducted in accordance with the Helsinki Declaration and was approved by the *Istituti Clinici Scientifici Maugeri* IRCCS Research Ethics Committee (Protocol no. 2279). 

We reviewed patient electronic medical records to collect the following data: baseline characteristics (including age, sex, BMI, and smoking history); asthma onset (childhood- or adult-onset asthma); history of atopy; self-reported respiratory symptoms (including chronic cough, symptom control, and episodes of acute exacerbation); health care utilization; pharmacological treatment; and comorbidities. Data were recorded anonymously on an electronic case report form. 

Adult-onset asthma was defined as asthma symptoms appearing at age ≥ 18 years, and childhood-onset asthma was defined as asthma symptoms appearing at age < 18 years.^16^ Atopy was defined by a positive skin prick test or the presence of specific IgE for inhalant allergens (> 0.1 kUA/L). Referral to a general practitioner, referral to a pulmonologist, or admission to the emergency department (with or without hospitalization) for worsening of symptoms in the 12 months preceding evaluation was reported as health care utilization and was scored from 0 to 3 (referral to a general practitioner = 0; referral to a pulmonologist = 1; admission to the emergency department without hospitalization = 2; and admission to the emergency department with hospitalization = 3). Disease control was assessed with the Asthma Control Questionnaire (ACQ).^17^

Lung function tests were performed with a Pony FX spirometer (COSMED srl, Rome, Italy) to assess FEV_1_ (in L and in % of predicted), FVC (in L and in % of predicted), and the FEV_1_/FVC ratio. All tests were performed in accordance with the American Thoracic Society/European Respiratory Society guidelines.^18^

In order to evaluate systemic and airway inflammation, we collected blood and induced sputum samples. Sputum was induced with inhaled hypertonic saline solution (at 4.5%) and processed with dithiothreitol, in accordance with European Respiratory Society guidelines.^19^ Patients presenting with concomitant clinically relevant respiratory diseases (e.g., COPD) and/or acute exacerbation in the month preceding the assessment were excluded. Patients with cough-variant asthma and those with cough-predominant asthma were not included in the analysis. 

### Statistical analysis

Qualitative variables were described as absolute and relative frequencies; quantitative data were summarized as means and standard deviations or medians and interquartile ranges, depending on their parametric or nonparametric distribution. Comparisons between ACC and ANCC for qualitative and quantitative variables were assessed with the chi-square test with Yates’ correction or Fisher’s exact test when appropriate, and the Student’s t-test or the Mann-Whitney test was used in case of parametric or nonparametric distribution, respectively. Values of p < 0.05 were considered statistically significant. Multivariate logistic regression analysis was performed to assess factors associated with ACC. Hierarchical logistic regression models with predictors added one at a time were examined in order to assess possible collinearity among the predictors. The predictors selected in the final model were based on numerical and clinical significance. Confounders such as gastroesophageal reflux disease and rhinitis were considered in the construction of the models. The quality of fit of the multiple logistic regression models was evaluated with the Hosmer-Lemeshow test. Odds ratios and 95% confidence intervals were presented. Data analysis was performed with the Predictive Analytics Software package, version 18.0 (SPSS Inc., Chicago, IL, USA). 

## RESULTS

Three-hundred and forty-four consecutive patients diagnosed with moderate-to-severe asthma were included in the present study (Table 1). The mean age was 57.7 ± 13.8 years; 202 (58.7%) were female; 139 (40.4%) were former smokers; 226 (65.7%) were atopic; 81 (23.7%) had childhood-onset asthma; and 27 (7.8%) reported professional exposure. The most common comorbidities were gastroesophageal reflux disease, in 144 (41.9%) of the patients; nasal polyposis, in 139 (40.7%); arterial hypertension, in 116 (33.7%); rhinitis, in 104 (30.2%); bronchiectasis, in 77 (22.4%); and obesity, in 61 (17.7%). 

**Table 1:** Comparison of demographic, epidemiological, and clinical characteristics between asthma patients with and without chronic cough.^a^

Variable	Total sample (N = 344)	Asthma patients with chronic cough (n = 124)	Asthma patients without chronic cough (n = 220)	p
Age, years	57.7 ± 13.8	58.9 ± 12.9	57.0 ± 14.3	0.235
Male sex	142 (41.3)	46 (37.1)	96 (43.6)	0.285
BMI, kg/m^2^	26.0 ± 5.1	26.5 ± 5.75	25.7 ± 4.74	0.163
Smoking status				
Nonsmoker	205 (59.6)	77 (62.1)	128 (58.2)	0.551
Former smoker	139 (40.4)	47 (37.9)	92 (41.8)	
Asthma onset				
Childhood	81 (23.7)	31 (25.2)	50 (22.8)	0.717
Adult	261 (76.3)	92 (74.8)	169 (77.2)	
Professional exposure	27 (7.8)	12 (9.7)	15 (6.8)	0.461
Atopy	226 (65.7)	80 (64.5)	146 (66.4)	0.819
Comorbidities				
OSA	54 (15.7)	17 (13.7)	37 (16.8)	0.544
Allergic rhinitis	104 (30.2)	33 (26.6)	71 (32.3)	0.329
CRSwNP	139 (40.4)	51 (41.1)	88 (40.0)	0.928
GERD	144 (41.9)	59 (47.6)	85 (38.6)	0.133
Ischemic heart disease	8 (2.3)	2 (1.6)	6 (2.7)	0.716
Atrial fibrillation	10 (2.9)	3 (2.4)	7 (3.2)	0.999
Arterial hypertension	116 (33.7)	46 (37.1)	70 (31.8)	0.381
Diabetes	23 (6.7)	7 (5.6)	16 (7.3)	0.722
Neoplasia	10 (2.9)	3 (2.4)	7 (3.2)	0.999
Bronchiectasis	77 (22.4)	28 (22.6)	49 (22.3)	0.999
Emphysema	35 (10.2)	10 (8.1)	25 (11.4)	0.432
CIRS 1	1.3 [1.3-1.5]	1.4 [1.3-1.6]	1.3 [1.2-1.5]	0.014*
CIRS 2	2 (2-3)	3 (2-4)	2 (2-3)	< 0.0001*
Nocturnal symptoms	37 (10.8)	22 (17.7)	15 (6.8)	0.003*
Wheezing	37 (10.8)	30 (24.2)	31 (14.1)	0.027*
Chest tightness	17 (4.9)	10 (8.1)	7 (3.2)	0.081
Dyspnea	96 (27.9)	48 (38.7)	48 (21.8)	0.001*
ICS dose				
Low	52 (15.1)	8 (6.5)	44 (20.0)	0.001*
Medium	113 (32.8)	38 (30.6)	75 (34.1)	0.593
High	179 (52.0)	78 (62.9)	101 (45.9)	0.004*
Antileukotriene	112 (32.6)	38 (30.6)	74 (33.6)	0.654
mOCS	53 (15.4)	23 (18.5)	30 (13.6)	0.291
LAMA	127 (36.9)	54 (43.5)	73 (33.2)	0.072
ACQ score	0.5 [0-1.4]	1 [0.17-1.67]	0.5 [0-1.17]	0.001*
Patients with exacerbations in the previous year	210 (61.0)	85 (68.5)	125 (56.8)	0.043*
Number of exacerbations in the previous year	1 [0-2]	2 [0-3]	1 [0-2]	0.011*
Health care utilization	193 (56.1)	88 (71.0)	105 (47.7)	< 0.0001*
General practitioner	24/193 (7.0)	8 (9.1)	16 (15.2)	0.285
Pulmonologist	151/193 (43.8)	73 (83.0)	78 (74.3)	0.201
Emergency department	14/193 (4.1)	5 (5.7)	9 (8.6)	0.623
Hospitalization	4/193 (1.2)	4 (2.1)	2 (2.3)	0.999
Pre-BD FEV_1_, L	2.42 ± 0.85	2.31 ± 0.79	2.47 ± 0.88	0.092
Pre-BD FEV_1_, % of predicted	87.2 ± 21.9	86.7 ± 22.8	87.5 ± 21.5	0.776
Pre-BD FVC, L	3.34 ± 1.06	3.20 ± 0.97	3.42 ± 1.09	0.069
Pre-BD FVC, % of predicted	95.9 ± 19.2	94.9 ± 19.8	96.6 ± 18.9	0.441
Pre-BD FEV_1_/FVC, % of predicted	71.8 ± 10.2	72.1 ± 9.89	71.6 ± 10.3	0.634
Blood leukocytes, 10^^9^/L	7.05 ± 1.81	7.27 ± 1.86	6.92 ± 1.77	0.087
Blood neutrophils, cells/µL	3,866.6 ± 1.35	4,011.7 ± 1.39	3,784.0 ± 1.33	0.142
Blood lymphocytes, cells/µL	2,136.3 ± 909.6	2,198.6 ± 703.3	2,100.9 ± 1.01	0.348
Blood eosinophils, cells/µL	301.1 [170.5-580.9]	322.9 [171.2-638.9]	287.5 [169.9-544.7]	0.414
Neutrophil-to-lymphocyte ratio	1.8 [1.36-2.37]	1.78 [1.33-2.33]	1.84 [1.36-2.37]	0.897
Induced sputum-inflammatory pattern				
Neutrophilic	77/297 (25.9)	30/100 (30.0)	47/197 (23.9)	0.543
Eosinophilic	135/297 (45.5)	46/100 (46.0)	89/197 (45.2)	
Mixed	48/297(16.2)	14/100 (14.0)	34/197 (17.3)	
Paucigranulocytic	37/297 (12.5)	10/100 (10.0)	27/197 (13.7)	
Induced sputum macrophages, %	14.8 [5.75-28.2]	14.4 [5.78-27.4]	15.5 [5.7-28.6]	0.837
Induced sputum neutrophils, %	50.7 [17.9-75.2]	50 [18.1-74.4]	50.7 [17.7-75.4]	0.756
Induced sputum eosinophils, %	6.9 [1.1-39.7]	5.8 [0.63-45.0]	7 [1.2-38.0]	0.767
Induced sputum lymphocytes, %	1 [0.3-1.8]	0.85 [0.2-2]	1.10 [0.3-1.75]	0.325

OSA: obstructive sleep apnea; CRSwNP: chronic rhinosinusitis with nasal polyps; GERD: gastroesophageal reflux disease; CIRS: Cumulative Illness Rating Scale; ICS: inhaled corticosteroid; mOCS: maintenance oral corticosteroids; LAMA: long-acting muscarinic antagonist; ACQ: Asthma Control Questionnaire; and BD: bronchodilator. ^a^Data expressed as n (%), mean ± SD, or median [IQR]. *Values of p < 0.05 were considered statistically significant.

With regard to lung function, mean FEV_1_% FVC%, and FEV_1_/FVC% were 87.2 ± 21.9, 95.9 ± 19.2, and 71.8 ± 10.2, respectively. 

Of a total of 344 asthma patients, 124 (36%) had CC (Table 1). Compared with patients without chronic cough, ACC had lower disease control (p = 0.001), higher dose of ICS (p= 0.004), higher number of exacerbations (p = 0.01), and health care utilization over the previous year (p < 0.0001) in those with chronic cough. The proportion of patients with nocturnal symptoms, wheezing and dyspnea was significantly higher in the ACC group (p = 0.003, p = 0.03, and p = 0.001, respectively), with higher Cumulative Illness Rating Scale (CIRS 1-2: p = 0.01 and p < 0.0001, respectively). The multivariate logistic regression analysis (Table 2) showed that dyspnea, nocturnal symptoms, CIRS 1, and health care utilization were factors independently associated with ACC. 

**Table 2:** Multivariate logistic regression analysis of factors associated with chronic cough in asthma patients.

Variable	b	SE	Wald	OR (95% CI)	p
Dyspnea	0.647	0.269	5.769	1.91 (1.13-3.24)	0.016*
Nocturnal symptoms	0.869	0.388	5.004	2.38 (1.11-5.10)	0.025*
CIRS-1	0.344	0.103	11.172	1.41 (1.15-1.73)	0.001*
Health care utilization	0.933	0.250	13.919	2.54 (1.56-4.15)	< 0.0001*

CIRS: Cumulative Illness Rating Scale. *Values of p < 0.05 were considered statistically significant.

No significant differences were observed between the two groups of asthma patients in terms of age, sex, BMI, age at asthma onset, smoking history, atopy, professional exposure, or comorbidities. In addition, there were no differences between the two groups regarding in lung function tests and levels of inflammatory biomarkers in blood or in sputum.

ACC and ANCC were further stratified by sputum eosinophil levels (< 3% vs. ≥ 3%) to investigate the role of eosinophilic bronchial inflammation (Table 3). In the ACC group, patients with bronchial eosinophilia were younger (p = 0.04), were more atopic (p = 0.007), and had worse lung function impairment (i.e., FEV_1_/FVC; p = 0.011) than those without bronchial eosinophilia. These results were not observed in the ANCC group. 

**Table 3:** Comparison of demographic, epidemiological, and clinical characteristics between induced sputum eosinophil levels (< 3% vs. ≥ 3%) in asthma patients with and without chronic cough.^a^

	Asthma patients with chronic cough (n =100)				Asthma patients without chronic cough (n = 197)		
Variable	IS eosinophils < 3% (n = 40)	IS eosinophils ≥ 3% (n = 60)	p	IS eosinophils < 3% (n = 74)	IS eosinophils ≥ 3% (n = 123)	p
Female sex	26 (65.0)	34 (56.7)	0.532	45 (60.8)	66 (53.7)	0.405
Age, years	62.5 ± 15.0	56.9 ± 11.5	0.041	56.4 ± 15.3	57.6 ± 13.8	0.585
BMI, kg/m^2^	25.7 ± 4.88	26.9 ± 5.95	0.256	26.4 ± 4.65	25.6 ± 5.02	0.300
Smoking status						
Nonsmoker	22 (55.0)	38 (63.3)	0.532	44 (59.5)	74 (60.2)	0.999
Former smoker	18 (45.0)	22 (36.7)		30 (40.5)	49 (39.8)	
Professional exposure	6 (15.0)	2 (3.3)	0.057	10 (13.5)	4 (3.3)	0.015
Atopy	17 (42.5)	43 (71.7)	0.007	52 (70.3)	78 (63.4)	0.407
Dyspnea	14 (35.0)	22 (36.7)	0.999	11 (14.9)	34 (27.6)	0.058
Asthma onset						
Childhood	7 (17.5)	16 (27.1)	0.385	19 (25.7)	23 (18.9)	0.343
Adult	33 (82.5)	43 (72.9)		55 (74.3)	99 (81.1)	
Pre-BD FEV_1_, % of predicted	86.2 ± 17.2	81.4 ± 19.1	0.205	87.7 ± 19.1	83.5 ± 19.5	0.137
Pre-BD FVC, % of predicted	90.1 ± 14.4	90.5 ± 15.7	0.903	94.6 ± 15.8	92.3 ± 16.1	0.324
Pre-BD FEV_1_/FVC	75.4 ± 7.82	70.6 ± 9.58	0.011	73.2 ± 10.8	70.6 ± 9.77	0.086
Blood eosinophils, cells/µL	202.5 [120.9 -349.8]	435.6 [191.1-678.9]	0.001	175.5 [95.4-251.1]	387.6 [249.2-640.7]	< 0.0001
Blood neutrophils, cells/µL	4,126.1 ± 1.40	3,794.4 ± 1.17	0.212	3,996.6 ± 1.58	3,697.9 ± 1.18	0.141
Blood lymphocytes, cells/µL	2,185.1 ± 128.9	2,143.2 ± 71.7	0.777	2,076.5 ± 679.6	2,081.3 ± 1.21	0.976
IS macrophages, %	19.6 [6.3-29.5]	13.05 [5.43-26.7]	0.224	20.5 [9.2- 39.4]	11.5 [5.4-23.6]	0.002
IS neutrophils, %	73.7 [60.0-89.8]	27.6 [10.0-58.9]	< 0.0001	70.2 [40.9- 84.0]	41.8 [13.8-65.8]	< 0.0001
IS lymphocytes, %	0.65 [0.2-1.9]	1.10 [0.23-2.10]	0.440	1.4 [0.8- 2.0]	0.9 [0.3-1.6]	0.010
ACQ score at baseline	0.75 [0.17-1.17]	1 [0.25-1.67]	0.089	0.5 [0-1.09]	0.33 [0-1.21]	0.784
Patients with exacerbations in the previous year	23 (57.5)	40 (66.7)	0.472	29 (39.2)	77 (62.6)	0.002
Number of exacerbations in the previous year	1.0 [0.0-2.0]	2.0 [0.0-2.75]	0.255	0.0 [0.0-1.0]	1.0 [0.0-2.0]	< 0.0001
Health care utilization	24 (60.0)	41 (68.3)	0.521	25 (33.8)	57 (46.3)	0.114

ACQ: Asthma Control Questionnaire; BD: bronchodilator; and IS: induced sputum. ^a^Data expressed as n (%), mean ± SD, or median [IQR]. *Values of p < 0.05 were considered statistically significant.

Interestingly, in the ANCC group, the proportion of exacerbating patients was significantly higher among those with bronchial eosinophilia (p = 0.002), whereas, in the ACC group this difference was not statistically significant (p = 0.5; Table 3). 

To further investigate the role of eosinophilic inflammation, patients were divided into two groups based on induced sputum eosinophils: 183/297 (61.6%) patients with airway eosinophilia ≥ 3% and 114/297 (38.4%) with eosinophils < 3%. A comparison between ACC vs ANCC was performed within each group. Among patients with eosinophils ≥ 3%, ACC patients showed higher ACQ score [median [IQR], 1 [0.25-1.67] vs. 0.33 [0.0-1.21]; p = 0.003) and health care utilization (68.3% vs. 46.3%; p = 0.005) in comparison to ANCC. In the eosinophils <3% group, ACC experienced more frequent exacerbations (median [IQR], 1.0 [0.0-2.0] vs. 0.0 [0.0-1.0]; p = 0.030), increased dyspnea (35% vs. 14.9%; p = 0.013), and increased health care utilization (60% vs. 33.8%; p = 0.007). 

## DISCUSSION

In our study, we divided moderate-to-severe asthma patients into two groups based on the presence of chronic cough as one of the symptoms, excluding patients with cough-variant asthma or cough-predominant asthma. 

Asthma patients with chronic cough were more severe in terms of the number of exacerbations per year, with worse symptom control (including nocturnal symptoms, wheezing, and dyspnea); needed additional health care utilization; and had higher cumulative illness rating scale (CIRS 1-2) in comparison with ANCC. However, there were no differences between the two groups of patients with regard to lung function impairment or airway and systemic inflammatory biomarkers, suggesting a hypothetical discrepancy between clinical and pathophysiological conditions in asthma patients with chronic cough. 

Dyspnea, nocturnal symptoms, CIRS 1, and health care utilization are factors independently associated with ACC. Indeed, it is known that cough and dyspnea have a worse impact on quality of life compared with other respiratory symptoms^10^ and, as observed in our study, uncontrolled asthma in ACC increases health care utilization. Studies^14^ ^,^ ^15^ ^,^ ^20^ ^-^ ^22^ have shown that asthma patients with cough have poorer asthma control and experience more frequent asthma exacerbations. In a six-month longitudinal analysis, patients with severe asthma and chronic cough had worse asthma control, more exacerbations, and worse quality of life as compared with patients without chronic cough.^15^ In a prospective cohort study,^14^ patients with asthma and chronic cough had more comorbidities, worse asthma control, increased moderate-to-severe exacerbations and health-care utilization in comparison with patients without chronic cough. Chronic cough, even when not associated with asthma, contributes to increased health-care service utilization and, consequently, higher health-care costs.^23^ ^-^ ^25^

Since there were no differences in terms of airway inflammation between ACC and ANCC, we stratified patients based on the presence of eosinophilic inflammation (sputum eosinophils ≥ 3%). ACC patients with eosinophilic airway inflammation were more atopic and had greater lung function impairment, whereas those without bronchial eosinophilia showed higher neutrophilic airway inflammation. This finding suggests that airway eosinophilia alone may be neither sufficient nor necessary to explain chronic cough in asthma. Furthermore, we found higher exacerbations in ANCC with sputum eosinophils ≥ 3% than in ANCC without sputum eosinophilia confirming the association between exacerbations and eosinophilic airway inflammation, as previously described.^26^ This difference was not found in asthma patients with chronic cough, underlying a more complex interaction between chronic cough, bronchial inflammation, and exacerbations. Nevertheless, the statistical difference that was observed only in the ANCC group was maybe due to the greater numerical difference between the subgroups (eosinophilic vs. noneosinophilic patients); the lower event rates; and the lower variability in the ANCC noneosinophilic subgroup. 

To further assess whether chronic cough contributes to increased clinical burden within the same inflammatory phenotype, we stratified individuals based on sputum eosinophils (≥ 3% and < 3%) and the presence or absence of CC. The results confirmed that ACC had a greater disease burden regardless of eosinophilic airway inflammation; among patients with eosinophils ≥ 3%, ACC showed higher ACQ score and greater health care utilization; in the eosinophils < 3%, ACC experienced more frequent exacerbations, increased dyspnea, and higher health care utilization. These results confirm that the role of bronchial inflammation is uncertain in ACC and alternative mechanisms such as mast cell mediator release,^27^ neutrophilic inflammation,^28^ hypersensitivity cough reflex,^29^ and comorbidities may play an important role in the genesis of cough. 

Although a recently published study^15^ described similar variables in severe asthma patients with and without chronic cough, our study is the first to analyze different patterns of bronchial inflammation assessed with from induced sputum in moderate-to-severe asthma patients not undergoing biologic therapy and therefore presenting with naïve inflammation. Further studies are needed to investigate this issue.

These data suggest that, despite the multifactorial nature of chronic cough, the presence of cough as a clinical symptom in asthma may help identify a subgroup of patients warranting referral to tertiary centers. Indeed, a “treatable traits” approach to chronic cough has been previously suggested, highlighting the importance of including cough in asthma assessment as part of personalized medicine^30^ to support phenotyping and the selection of patients eligible for biological treatment. 

The main limitation of the present study is its retrospective and single-center design. Nevertheless, the study reported the vast majority of biological and physiological characteristics of asthma patients in real life. 

Our preliminary data suggest that asthma patients with chronic cough are prone to worse disease severity and worse symptom control, and should therefore be carefully evaluated in asthma referral centers. Various validated questionnaires such as the Asthma Control Test,^31^ the ACQ,^17^ and the Asthma Quality of Life Questionnaire^32^ have been introduced in clinical practice and in clinical trials as asthma management outcomes. Nevertheless, these tools do not fully mirror the specific impact of cough in asthma control, only general population questionnaires such as the Leicester Cough Questionnaire being currently available for this purpose.^33^ Further studies are needed to evaluate whether routine administration of specific cough questionnaires in asthma clinics can improve asthma management. 
